# Mealtime Behaviors in Nursing Home Residents With Dementia: Validating Clinically Practical Scales

**DOI:** 10.1093/geroni/igaf122.1337

**Published:** 2025-12-31

**Authors:** Wen Liu, Yelena Perkhounkova, Maria Hein

**Affiliations:** Penn State University, University Park, Pennsylvania, United States; University of Iowa, Iowa City, Iowa, United States; University of Iowa, Iowa City, Iowa, United States

## Abstract

Optimizing mealtime behaviors (i.e., positive behaviors, functional impairments, resistive behaviors) is critical to improve enjoyment of mealtimes, nutrition, and function in residents with dementia. Few tools are valid and clinically practical to measure resident mealtime behaviors. We adapted the Cue Utilization and Engagement in Dementia (CUED) video-coding scheme, a novel, valid, yet resource-intensive tool, into three observational scales to assess resident mealtime behaviors and examined their structural validity. N = 425 videos of dyadic mealtime interactions capturing n = 44 residents in 10 nursing homes were coded using CUED. Coded resident mealtime behaviors (i.e., items) were categorized based on frequency: 0 (not observed), 1 (observed 1-2 times), and 2 (observed ≥3 times). Some behaviors were combined based on content relevance, redundancy, and distributions, resulting in 8 items for positive behaviors, 6 items for functional impairments, and 8 items for resistive behaviors. A total of 20 random splits of 425 videos were created for exploratory (EFA) and confirmatory factor analyses (CFA) of each item set. EFA supported 1-factor structure for the three scales. Based on model fit and conceptual interpretability, three items were selected for the positive behaviors scale (Cronbach alpha=.65), four items were selected for the functional impairments scale (alpha=.38), and four items were selected for the resistive behaviors scale (alpha=.44). The 3-item positive behaviors scale, 4-item functional impairment scale, and 4-item resistive behaviors scale were developed for pragmatic use in research and practice. Future testing is needed through direct observations of mealtime care in nursing homes to establish validity and clinical utility.

